# RETRACTION: Puerarin 6″‐O‐xyloside suppressed HCC via regulating proliferation, stemness, and apoptosis with inhibited PI3K/AKT/mTOR

**DOI:** 10.1002/cam4.70301

**Published:** 2024-10-10

## Abstract

**Retraction:** L. Li, J.‐D. Liu, G.‐D. Gao, K. Zhang, Y.‐W. Song, and H.‐B. Li, “Puerarin 6″‐O‐xyloside Suppressed HCC via Regulating Proliferation, Stemness, and Apoptosis with Inhibited PI3K/AKT/mTOR,” *Cancer Medicine* 9, no. 17 (2020): 6399–6410. https://doi.org/10.1002/cam4.3285.

The above article, published online on 21 July 2020, in Wiley Online Library (wileyonlinelibrary.com), has been retracted by agreement between the journal Editor in Chief, Stephen Tait, and John Wiley & Sons Ltd. A report submitted by a third party noted image duplications with other articles by different author groups. Specifically, the report found image duplications with other published articles in Figures 2A, 2C, 3A, 4A, 5D, and 6A. The retraction has been agreed to because the evidence of image duplication across different articles, each of which describes different experimental conditions, fundamentally compromises the editors' confidence in the conclusions presented. The authors did not respond to our notice regarding the retraction.

**Retraction:** L. Li, J.‐D. Liu, G.‐D. Gao, K. Zhang, Y.‐W. Song, and H.‐B. Li, “Puerarin 6″‐O‐xyloside Suppressed HCC via Regulating Proliferation, Stemness, and Apoptosis with Inhibited PI3K/AKT/mTOR,” *Cancer Medicine* 9, no. 17 (2020): 6399–6410. https://doi.org/10.1002/cam4.3285.

The above article, published online on 21 July 2020, in Wiley Online Library (wileyonlinelibrary.com), has been retracted by agreement between the journal Editor in Chief, Stephen Tait, and John Wiley & Sons Ltd. A report submitted by a third party noted image duplications with other articles by different author groups. Specifically, the report found image duplications with other published articles in Figures 2A, 2C, 3A, 4A, 5D, and 6A. The retraction has been agreed to because the evidence of image duplication across different articles, each of which describes different experimental conditions, fundamentally compromises the editors' confidence in the conclusions presented. The authors did not respond to our notice regarding the retraction.

